# Finite element analysis of dynamic changes in spinal mechanics of osteoporotic lumbar fracture

**DOI:** 10.1186/s40001-022-00769-x

**Published:** 2022-08-06

**Authors:** Jianwen Yan, Zhong Liao, Yafang Yu

**Affiliations:** 1 Fuzhou Second Hospital, Fuzhou, China; 2 Fujian Medical University Union Hospital, Fuzhou, China

**Keywords:** Finite element analysis, Osteoporosis, Thoracolumbar fracture, Spinal mechanics, Clinical application study

## Abstract

**Aim:**

This study aims to explore the effects of finite element biomechanical properties of different methods in the treatment of osteoporotic thoracolumbar fractures.

**Methods:**

Based on the ultra-thin computed tomography scan data of a volunteer’s thoracolumbar spine, the finite element method was used to simulate the treatment of osteoporotic thoracolumbar fracture. Spiral computed tomography scanning was used to obtain images of the thoracolumbar region, which was then imported into Mimics software to obtain the three-dimensional geometric model. The finite element model of normal T_11_ – L_2_ segment was established by finite element software Abaqus and the validity of the model loading was verified. The finite element model of T_11_ vertebral compression fracture was established based on normal raw data. The clinical overextension reduction manipulation was simulated by different treatment methods and the changes in stress and displacement in different parts of injured vertebrae were analyzed.

**Results:**

An effective finite element model of T_11_–L_2_ segment was established. The maximum stress, axial compression strength, axial compression stiffness, and transverse shear stiffness were significantly better in the percutaneous kyphoplasty and percutaneous vertebroplasty treatment group than in the conservative treatment group and open treatment group (*P* < 0.05). Additionally, there was no significant difference between the open treatment group and conservative treatment group, or between the PKP and PVP treatment group.

**Conclusion:**

Percutaneous vertebroplasty and percutaneous kyphoplasty not only met the requirements of normal functional kinematics of thoracolumbar spine, but also restored the stability of thoracolumbar spine. They had good biomechanical properties and remarkable application effects. The application of finite element analysis can help select a scientific, reasonable, and effective treatment scheme for the clinical diagnosis and treatment of osteoporotic thoracolumbar fractures.

## Introduction

Osteoporosis is one of the common basic diseases in the elderly with calcium loss in the body and bone mass reduction which is more prone to cause fracture and occur in the thoracolumbar vertebrae [1, 2]. Osteoporosis is a systemic skeletal disease, the occurrence and development of which is the result of the comprehensive action of many systems, such as nerve, endocrine, immunity, reproduction, musculoskeletal, and so on. It is characterized by decreased bone mass, bone microstructure degeneration, increased bone brittleness, pain, lumbar and knee soreness, spinal deformation, and other symptoms, resulting in bone brittleness and fracture susceptibility. Osteoporosis has been demonstrated as one of the main causes of fracture in the elderly in the clinic [3]. The main clinical manifestations of dyskinesia, low back pain, and loss of self-care ability and labor ability seriously affect the quality of life of the patients [4–6]. With the aging population, the incidence of osteoporotic thoracolumbar compression fractures in the elderly has increased year by year. It remains one of the major killers of the health of the elderly [7]. In addition, there is a lack of early diagnosis of osteoporotic lumbar fracture and no unified standard to systematically evaluate the severity of osteoporotic lumbar fracture. The clinical diagnosis of an osteoporotic vertebral compression fracture (OVCF) is challenging and requires detailed assessment using comprehensive imaging methods [8]. Due to serious morbidity and potential mortality, it has been increasingly recognized as an important medical problem. Seeking prevention, diagnosis, and treatment of osteoporotic lumbar fracture is still a major clinical problem. According to the epidemiological survey conducted by the China National Health Commission, about 19.2% of people over the age of 50 and 32.0% of people over 65 suffer from osteoporosis, with a higher incidence in postmenopausal women than in men [16]. Osteoporotic thoracolumbar fracture is one of the common complications of osteoporosis. It is estimated that the prevalence rate of vertebral fractures is about 16–21% in different regions of the world, with 30–50% of patients experiencing back pain, kyphosis, acute vertebral dyskinesia, and neurological dysfunction caused by spinal cord injury [17]. The main risk factors of osteoporotic vertebral fracture include rheumatoid arthritis, type 2 diabetes mellitus, the use of glucocorticoid and immunosuppressant, low body mass index, and so on, while the strongest risk factors are advanced age and bone mineral density [18–20].

The current treatment of osteoporotic thoracolumbar fracture is mainly divided into conservative treatment and minimally invasive surgical treatment. Conservative treatment includes bed rest, pain relief, anti-osteoporosis, and brace fixation, but with a long treatment cycle and imperfect effect [9–11]. With the development of minimally invasive technology, vertebroplasty has become the main treatment for osteoporotic thoracolumbar fractures with good results. However, the patients' bone fractures heal slowly, and there is a risk of nerve injury, pulmonary embolism, and other complications caused by leakage of filling materials [12–14]. Unfortunately, the expensive and complex method limits its clinical application.

Based on this, this study used conservative treatment, percutaneous vertebroplasty (PVP), percutaneous kyphoplasty (PKP), and open surgery to treat patients with thoracolumbar fractures through finite element analysis given biomechanical properties. The biomechanical axial compression strength and axial compression stiffness (EF) of each group were compared, to make the best qualitative and quantitative treatment plan for the selection of clinical prevention and treatment methods.

## Materials and methods

### Modeling research object

One volunteer was selected (weight 70 kg, height 172 cm) and the multi-layer spiral computed tomography (CT) scan (GE, USA) was used as the basis for building a perfect model. The volunteers without spinal deformity, tumors, and other spinal diseases were included in the experiment after the approval of the ethics committee of the hospital and the informed consent of the patients or their families. The general data of patients were collected, including gender, age, weight, height, body mass index, etiology and course of diseases. Inclusion criteria were as follows: (A) osteoporotic thoracolumbar fracture diagnosed by dual energy X-ray bone mineral density and imaging; (B) fracture compression 1/3–1/2; (C) no history of any treatment; (D) the course of disease was more than 3 months; (E) patients agreed and signed the informed consent form. Exclusion criteria were as follows: A. no osteoporotic traumatic fracture; B. complicated with other organ diseases; C. suffering from malignant tumors; D. patients with mental illness; E. patients with spinal cord injury or nerve injury. The CT scanning data of thoracolumbar vertebrae of volunteers were obtained. Patients were placed in the supine position, then spiral CT was used to scan the spine of T11–L2 continuously with 0.62 mm spacing, and the CT images of T11–L2 segments in Dicom format were obtained. 120 kV 125 mA, layer thickness 0.62 mm, and layer spacing 0.62 mm were set for the scan.

Ten patients with osteoporotic thoracolumbar fracture treated with different treatment methods (conservative treatment, open surgery, PKP, and PVP) in the Department of orthopaedics of our hospital from January 2021 to August 2022 were selected as the research object. The patients had no spinal column deformity, tumors, and other spinal diseases. The patients were enrolled in the experiment after approval by the ethics committee of our hospital and informed consent of the patients or their family members. The general data of patients were collected, including gender, age, weight, height, body mass index, etiology and course of disease. There was no significant difference in baseline characteristics between the 4 groups (*P* > 0.05) (Table 1). Patients were taken in the supine position, and the thoracolumbar T10 to L2 segment was scanned continuously with spiral CT at an interval of 0.62 mm to obtain the CT images in DICOM format, which were then recorded and stored in the CD-ROM. Patients were given various loads to record and compare the dynamic distribution of spine deformation and stress under the load.

**Table 1:** Comparison of the baseline of the patients in the 4 treatment groups at baseline

Characteristic	Conservative treatment group (*n *= 10)	Open surgical treatment group (*n* = 10)	PKP group (*n* = 10)	PVP group (*n *= 10)
Gender, male/female	5/5	4/6	6/4	5/5 0.973
Age, years	65.1 ± 4.94	61.5 ± 3.55	65.4 ± 4.51	66.3 ± 4.87
Bone mineral density	−2.78 ± 0.36	−2.57 ± 0.59	−2.81 ± 0.61	−2.85 ± 0.20
Level of fractured vertebrae (T12/L1/L2)	3/3/4	2/4/4	3/4/3	3/4/3

Data were presented as mean ± standard deviation or number (*P* > 0.05)

The finite element method was used to compare the efficacy of four treatments for osteoporotic thoracolumbar fractures. The three-dimensional finite element model of thoracolumbar fracture was established by Mimics17.0 Software (Materialise, Belgium), SolidWorks 2015 (Dassault systems SA, USA), ABAQUS 2016 (Dassault systems SA, USA), and other software. And four different treatment methods were implanted, in which the first was to simulate the conservative treatment, the second was to simulate the open surgical treatment, the third was to simulate the PKP, and the fourth was to simulate the PVP.

### Instrumentation and analysis software

Mimics17.0 Software (Materialise, Belgium); Geomagic Studio11 (Geomagic, USA); Solid Works 2015 (Dassault systems S.A, USA); ABAQUS 2016 (Dassault systems S.A, USA); Multi-layer spiral CT (GE, USA).

### Inclusion criteria

The patients with thoracolumbar fracture were diagnosed by magnetic resonance imaging, CT, or X-ray examination; the bone mineral density *T* value of less than-2.5 by the dual energy X-ray absorptiometry represented osteoporosis; fracture compression 1/3 or 1/2; the vertebrae, ligaments, and facet joints were intact; no congenital malformation or tumor in the patients; patients or their families signed the informed consent and voluntarily participated in treatment.

### Exclusion criteria

Exclusion of compression fractures caused by multiple myeloma; complicated with the pedicle fracture and spinal cord injury; complicated with severe heart, brain, liver, kidney, endocrine, malignant tumors, and other diseases; abnormal blood coagulation or congenital blood system diseases.

### Treatment method

#### Conservative treatment

The patients were treated conservatively by resting on the hard bed, receiving the corresponding painkillers according to the degree of pain, stopping the painkillers when the pain was tolerable or disappeared completely, and receiving the anti-osteoporosis vitamin D_3_ treatment. Patients wore the waist brace for functional exercise 2 months later.

#### Open surgical treatment

Patients were placed in the prone position after anesthesia, and the injured vertebra was taken as the central longitudinal incision to expose the pedicle of the injured vertebra, as well as the upper and lower adjacent vertebrae after disinfection. The pedicle screw was screwed in advance and then exited. The bone cement of the preparation number was injected into the PVP tube. After thoroughly dispersing the bone cement, it was screwed into the pedicle screw and solidified. After total laminectomy and decompression, the connecting rod of the spinal internal fixation device was installed to extend the reduction. The stitches were removed 14 days after operation and the patients wore the lumbar brace to get out of bed 2 weeks later.

##### PKP

The skin was cut open after disinfection. The guide needle was entered through the pedicle approach to 5 cm of the posterior wall of the vertebral body along which the dilation tube channel was entered followed by the balloon dilator. Then polymethyl methacrylate was injected and left to solidify. After the operation, the patient went to the pillow and lay flat for 6 h, then wore a waist brace to get out of bed.

##### PVP

The patients were placed in the prone position and gently manipulated under the guidance of mobile C-arm X-ray machine (model: wi294489) after local anesthesia to make the shape of bilateral vertebral arch symmetrical. After disinfection, the needle was punctured to the third place of the vertebral body and the bone cement was slowly injected into the injured vertebra with a pressure syringe. The distribution of bone cement was observed by C-arm machine at any time. The patient was transferred to the ward 30 min after surgery. Patients went to the pillow and lay flat for 6 h, then wore a waist brace to get out of bed.

### Observational indexes

① The maximum stress value of the four groups of models; ② the stress changes of the facet joints in different intervertebral spaces under different movements after the static load of the model; ③ the axial compression strength of four treatment methods for thoracolumbar fracture; ④ the stiffness of four treatment methods for thoracolumbar fracture.

### Statistical analysis

Statistical analyses were carried out using SPSS 23.0 (IBM Corporation, Armonk, NY), and a *t*-test was used to compare the biomechanics of T_11_ – L_2_ segments in normal and fracture states. With normal biomechanics as the control group, the biomechanics of each group after different treatments were analyzed by one-way analysis of variance and multiple comparison least significant difference test (*P* < 0.05). *P* < 0.05 was considered statistically significant.

## Results

### *T*_*11*_*–L*_*2*_* segment finite element model*

**Fig. 1:**
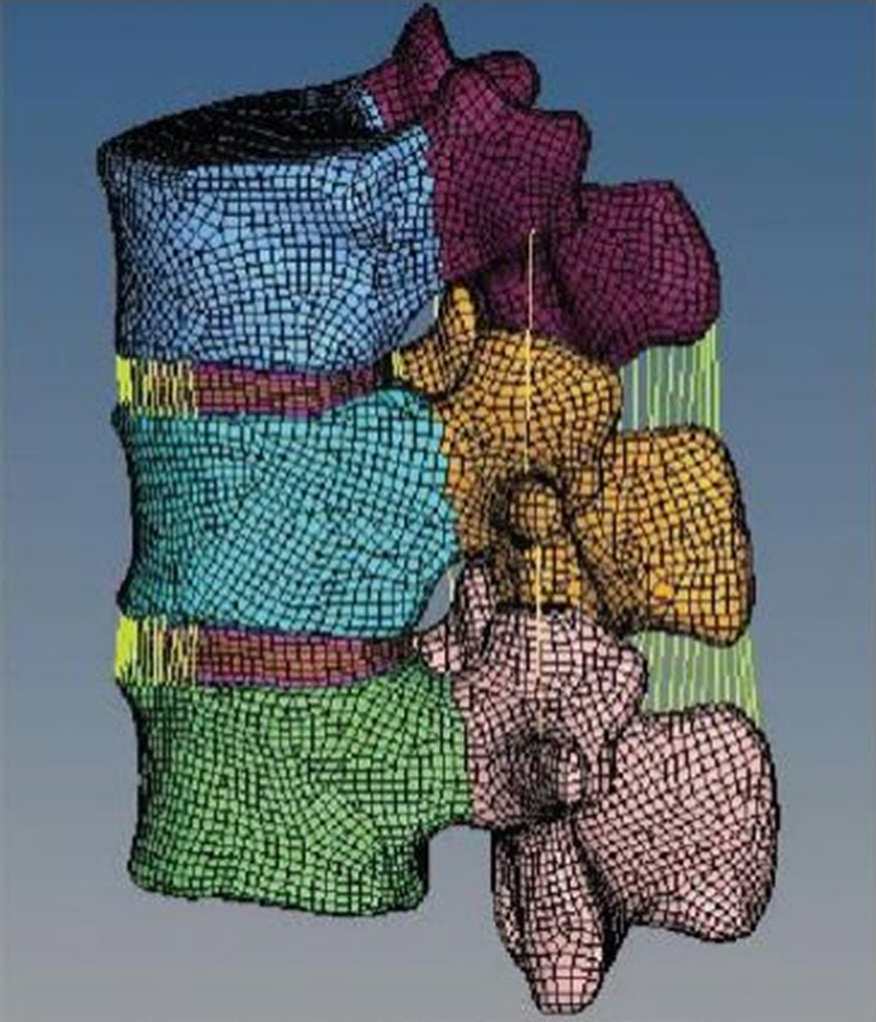
The segment finite element model

The model included cortical bone, cancellous bone, posterior longitudinal ligament, anterior longitudinal ligament, facet joint, fibrous annulus, nucleus pulposus, ligament flavum, interspinous ligament, articular capsule ligament, supraspinous ligament, and so on. As shown in Fig. 1, the model had 209,026 elements and 61,738 nodes.

### Verification of the validity of the model

**Fig. 2:**
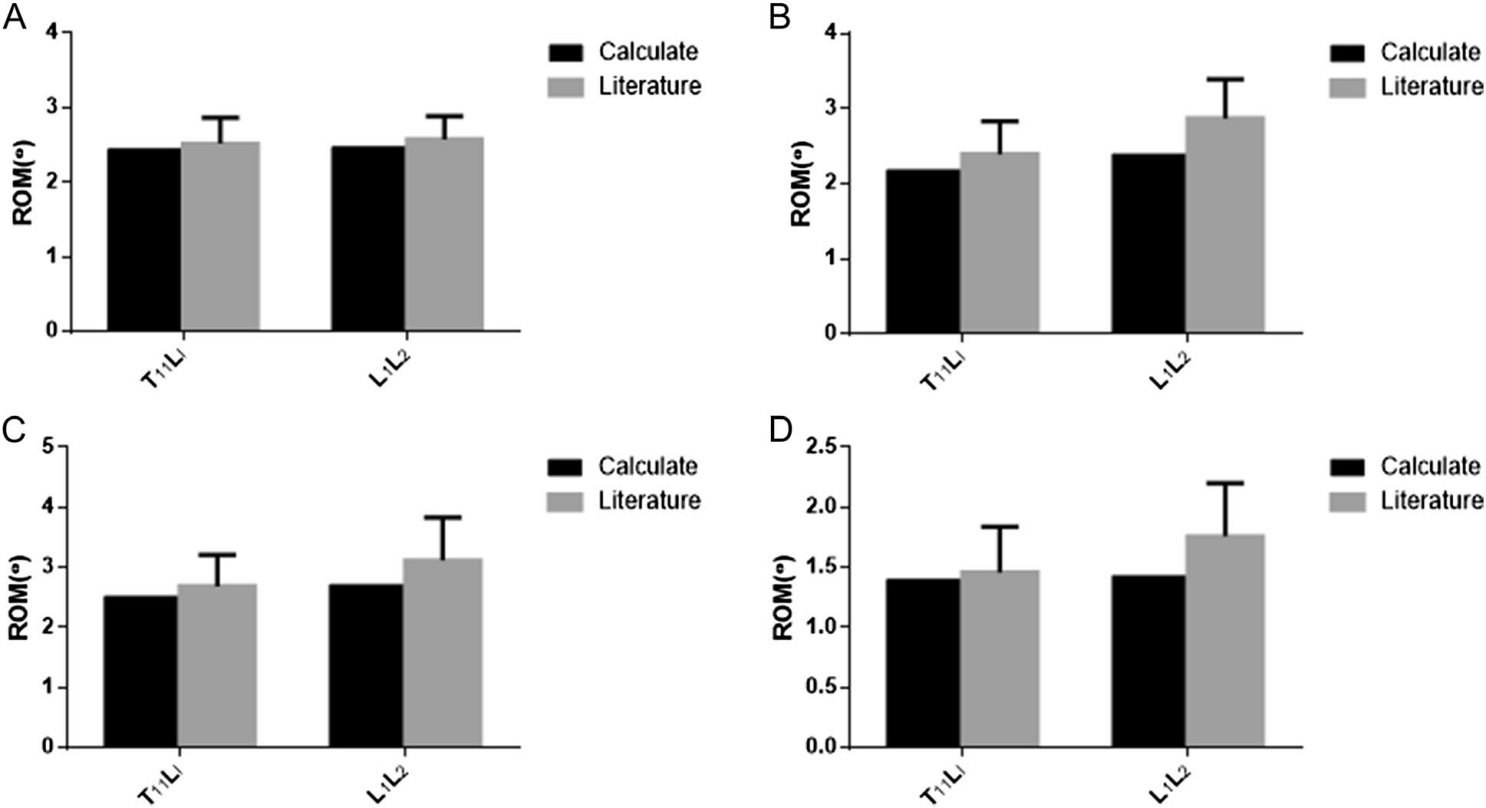
Comparison of ROM in the model and literature. **A** Anteflexion ROM; **B** backward extension ROM; **C** side bend ROM; **D** rotate ROM

The range of motion (ROM) of T_11_– L_2_ segment was as follows: T_11_ – L_1_ flexion (2.42°), L_1_– L_2_ flexion (2.39°), T_11_ – L_1_ dorsal extension (2.31°), L_1_ – L_2_ dorsal extension (2.48°), T_11_ – L_1_ lateral bending (2.55°), L_1_– L_2_ lateral bending (2.60°), T_11_ – L_1_ axial rotation (1.38°), L_1_– L_2_ axial rotation (1.39°). As shown in Fig. 2, the ROM of the model was similar to that of the literature [15].

With the gradual increase of stress, the comprehensive stress changes of the seven working conditions were the largest in the conservative treatment group. It indicated that if the stress concentration of the thoracolumbar vertebrae increased significantly after the treatment of thoracolumbar fractures with this method, the risk of re-fracture after continued compression remained high (Table 2). The comprehensive stress change of 7 working conditions in the open surgery was smaller than that in the conservative treatment group. It suggested that if the stress concentration of thoracolumbar vertebrae increased significantly, the risk of re-fracture decreased after the continued compression. The comprehensive stress changes of the seven working conditions of the model were small after the PKP and PVP treatment. It implied that if the stress concentration of thoracolumbar vertebrae increased significantly after this method for the treatment of thoracolumbar fracture, the risk of re-fracture was smaller.

**Table 2:** Comparison of maximum stress values of four groups of models ($\overline{\mathrm{x}}\pm \mathrm{s }$, mPA, *P* < 0.01)

Working condition	Conservative treatment group	Open surgical treatment group	PKP group	PVP group
Vertical load	11.97 ± 4.29	11.61 ± 3.26	10.87 ± 1.98	10.67 ± 5.17
Anteflexion	42.24 ± 7.92	41.87 ± 9.03	35.10 ± 6.09	37.27 ± 2.92
Extension	42.72 ± 6.28	41.64 ± 7.35	39.79 ± 8.03	38.07 ± 5.58
Left flexion	54.49 ± 8.01	42.72 ± 3.65	36.24 ± 3.97	37.31 ± 4.12
Right flexion	52.63 ± 6.77	41.70 ± 7.02	35.03 ± 2.55	38.20 ± 2.84
Left rotation	16.17 ± 5.11	13.22 ± 1.60	13.24 ± 2.04	13.18 ± 2.13
Right rotation	14.42 ± 2.15	11.74 ± 1.04	11.55 ± 0.99	11.72 ± 1.79

### Axial compression strength of four treatment methods for thoracolumbar fracture

**Fig. 3:**
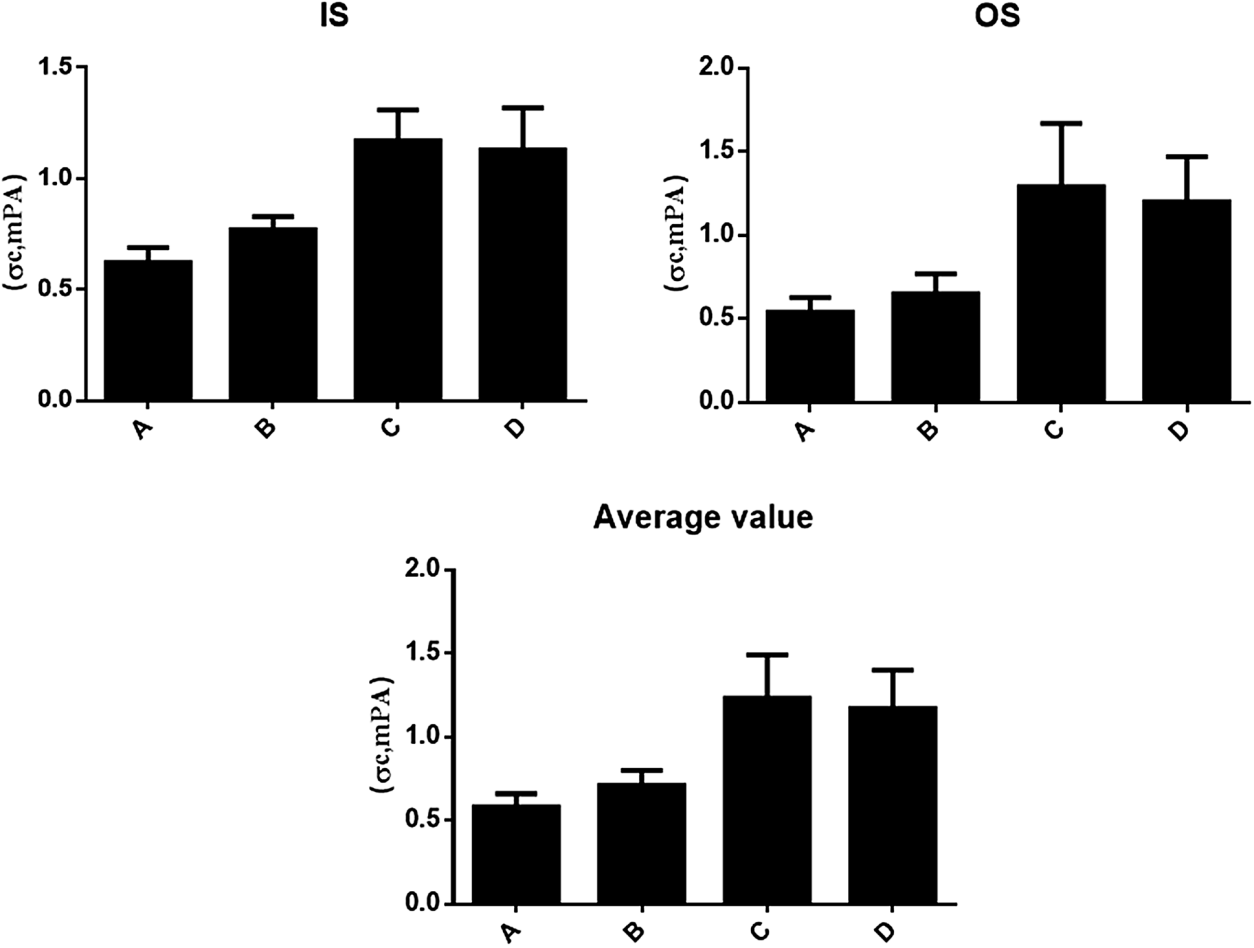
Axial compression strength of four treatment methods for thoracolumbar fracture (σc, $\overline{\mathrm{x}}\pm \mathrm{s }$, mPA). **A** Conservative treatment group; **B** open surgical treatment group; **C** PKP group; **D** PVP group

The thoracolumbar strength refers to the ability of thoracolumbar vertebrae to resist destruction under load, representing the strength of thoracolumbar vertebrae after different treatments. Conservative treatment, open treatment, PKP treatment, and PVP treatment were all used to treat osteoporotic thoracolumbar fractures. Notably, there was no significant difference between the PKP group and PVP group, but the axial compression strength of the PKP and PVP treatment group was significantly higher than that of the conservative treatment group (*t* = 3.043, *P* = 0.047). As shown in Fig. 3, the axial compression strength of the PKP and PVP treatment group was significantly higher than that of the conservative treatment group (*t* = 4.721, *P* = 0.041) And the therapeutic effects of the PKP and PVP group were better with no significant difference between two groups.

### Stiffness of four treatment methods for thoracolumbar fracture

**Fig. 4:**
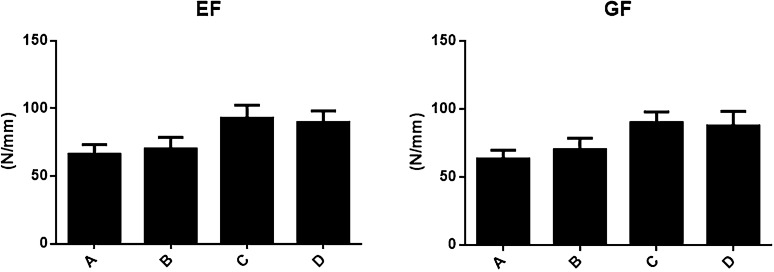
Stiffness of four treatment methods for thoracolumbar fracture (N/mm, $\overline{\mathrm{x}}\pm \mathrm{s }$) **A** Conservative treatment group; **B** open surgical treatment group; **C** PKP group; **D** PVP group

The axial stiffness of thoracolumbar vertebrae represents the ability to resist axial deformation of thoracolumbar vertebrae under load, which is one of the mechanical indexes of the stability of thoracolumbar internal fixation. Osteoporotic thoracolumbar fractures were treated with open treatment, conservative treatment, PKP treatment, and PVP treatment. As shown in Fig. 4, the groups with highest to lowest axial compression stiffness (EF) were PKP treatment, PVP treatment, open treatment, and conservative treatment, respectively. And there was no significant difference in the axial compression strength between the PKP group and PVP group (*t* = 3.482, *P* = 0.057; *t* = 3.121, *P* = 0.061). However, the axial compression strength of PKP and PVP group was significantly higher than that of conservative treatment group (*t* = 4.223, *P* = 0.046). The transverse shear stiffness (GF) of open treatment group, PKP group, and PVP group was similar to that of conservative treatment group, and that of PKP treatment group was similar to that of PVP treatment group with no significant difference (*t* = 3.081, *P* = 0.052; *t* = 3.742, *P* = 0.057). The axial compression strength of PKP and PVP group was significantly higher than that of conservative treatment group (*t* = 4.043, *P* = 0.044). Moreover, the effect of PKP and PVP treatment was better with no significant difference between two groups.

## Discussion

At present, osteoporotic fracture is considered to be an important public health problem that not only increases morbidity and mortality in the elderly, but also incurs important economic costs. In addition, osteoporotic lumbar fracture often causes spinal cord injury, which is the main cause of disability, reduced quality of life, and even death in the elderly [1]. Thoracolumbar vertebra refers to T_11_–L_2_, which is a special segment of the spine. Because of the special anatomical structure located between the fixed thoracic vertebrae and the active lumbar vertebrae and the stage of transition from the physiological kyphosis of the thoracic vertebrae to the physiological protrusion of the lumbar vertebrae, it is easy to cause injury under stress. With aging, the thoracolumbar vertebrae tend to show progressive degenerative changes that increase fibrosis and uneven distribution of compressive stresses, thus exposing some parts of the vertebral body to high-stress concentrations. The trabecular bone adjacent to the endplate can then adapt to the changed force distribution according to Wolff's law, leading to observed changes in bone architecture and density [21]. Osteoporosis affects the spine in another way. Severe osteoporosis affects the shape of the bone, resulting in a change in the height of the lumbar vertebrae and shifting the arches of the adjacent vertebrae closer. According to the spinal three-column theory , the lumbar vertebrae play a major role in spinal mechanics as a cushion and stress disperser [22]. When osteoporosis occurs in the lumbar vertebra, the way this vertebra bears the transmission force also changes. Osteoporotic vertebral compression fracture reduces the strength of the vertebral body and loses the stability of the spine. Meanwhile, the fretting of the fracture site may stimulate the peripheral nerves of bone marrow and periosteum, inducing pain. Therefore, it is critical to explore the biomechanics of this section. From the perspective of biomechanics, the purpose of thoracolumbar fracture treatment is to restore the strength, stiffness, and stability of the thoracolumbar spine, and to achieve the bony healing of the thoracolumbar vertebrae. The biomechanical experimental results showed that PKP and PVP might meet this requirement for osteoporotic thoracolumbar fractures. The biomechanical properties of the model were significantly improved after the PKP and PVP treatment, which met the requirements of normal functional kinematics of thoracolumbar vertebrae and restored the stability of thoracolumbar vertebrae.

There are many biomechanical research methods, and one of the new biomechanical research methods, finite element analysis, has shown its unique superiority since its application. However, this method is still not very mature with many defects, such as time-consuming, data loss, not all lifelike appearance, and so on [23]. Finite element models need to be validated with experimental data to make sure that they can represent the complex mechanical and physiological behavior of normal, injured, and stabilized spinal segments. With the unremitting efforts of many scholars over years, the clinical value of the finite element model of thoracolumbar spine is increasing, with more and more realistic shapes and biological characteristics that are getting closer and closer to the real law of motion of the human body. Under the environment with the above-mentioned advantages, this study took the bone structure data of a patient with the osteoporotic thoracolumbar fracture and used the ultra-thin CT scanning technology to obtain the sectional image of T11–L2. The authenticity of the original data needed for modeling was high. Then, the CT thin layer scanning image was read into the Mimics software to establish the geometric model. The Mimics software automatically assigned materials according to the CT value of the original model data and made the assignment accurate and fast, to ensure the high accuracy of the model in this study. Once validated, finite element models can be used to study the effects of different bone structures or intervertebral discs on the biomechanical behavior of spinal segments.

PKP has been widely used in the treatment of thoracolumbar fractures for fracture reduction. This method can not only restore vertebral body height and correct vertebral kyphosis deformity, but also reduce pain, the leakage rate of bone cement, and complications, such as nerve injury and pulmonary embolism [24]. PKP has been reported to be clinically valuable for the treatment of thoracolumbar fracture because of its short operation time, less trauma, low cost, short radiotherapy time, and so on [25]. This study revealed that there was no significant difference in the peak stress and displacement of the model between PKP and PVP treatment. Importantly, the difference was similar and the peak difference was small.

This study further clarified the prospective application of finite element analysis in osteoporotic thoracolumbar fractures. The commonly used static fixation simulation method was used to simulate the biomechanics of different treatment methods for osteoporotic thoracolumbar fracture in this study. In reality, the thoracolumbar spine is mainly subjected to the dynamic force in the course of action life, while muscles and other soft tissues also have a certain impact on the force of the thoracolumbar vertebrae. Therefore, this study only reflects the stress of the thoracolumbar vertebrae under a certain action, and the dynamic analysis of the musculoskeletal system needs to be combined at a later stage to provide more accurate and practical simulation results for the clinic. In this study, finite element technology was used to simulate the changes in the spinal mechanics environment of osteoporotic thoracolumbar fractures, exploring the effects of different treatment methods on the dynamic changes of spinal mechanics and the application of finite element analysis in the dynamic changes of spinal mechanics of osteoporotic thoracolumbar fractures. It may help select a scientific, reasonable, and effective treatment scheme for clinical diagnosis and treatment of osteoporotic thoracolumbar fractures.

In conclusion, PvP and PKP not only met the requirements of normal functional kinematics of thoracolumbar spine, but also restored the stability of thoracolumbar spine. They had good biomechanical properties and remarkable application effects. The application of finite element analysis can help select a scientific, reasonable, and effective treatment scheme for the clinical diagnosis and treatment of osteoporotic thoracolumbar fractures.

## Data Availability

The datasets used or analyzed in this study are available from the corresponding author after a reasonable request.
